# Macrophage Proteomic Profiling Reveals Divergent TLR4-Dependent and -Independent Responses to Kdo_2_-Lipid A and Lipid IVa

**DOI:** 10.3390/life16050753

**Published:** 2026-05-01

**Authors:** Jiraphorn Issara-Amphorn, Jenna L. Schoonmaker, Clinton Bradfield, Sung Hwan Yoon, Iain D. C. Fraser, Aleksandra Nita-Lazar

**Affiliations:** 1 Functional Cellular Networks Section, Laboratory of Immune System Biology, National Institute of Allergy and Infectious Diseases, National Institutes of Health, Bethesda, MD 20892-1892, USA; 2 Signaling Systems Section, Laboratory of Immune System Biology, National Institute of Allergy and Infectious Diseases, National Institutes of Health, Bethesda, MD 20892-1892, USA

**Keywords:** macrophages, non-canonical inflammasome, intracellular pathogen, Toll-like receptor 4, LPS signaling pathway, TLR4-independent, TLR4-dependent, Caspase-11, Kdo_2_-lipid A, lipid-IVa

## Abstract

Macrophages harness pattern recognition receptors (PRRs) to detect conserved bacterial components and mount effective immune responses. Many Gram-negative bacteria modify their lipid A structures to limit recognition by Toll-like receptor 4 (TLR4) and cytosolic Caspase-11 lipopolysaccharide sensors. One common evasion strategy is to reduce the lipid A acylation state from hexa- to tetra-acylation. This alteration can limit binding to receptors and dampen subsequent immune signaling responses, yet the proteomic alterations associated with this altered immunogenicity remain incompletely understood. Here, we systematically profiled proteomic alterations induced by extracellular or transfected hexa-acylated Kdo2-lipid A (Kdo2) and tetra-acylated lipid-IVa (IVa) to assess TLR4-dependent, TLR4-independent, and non-canonical inflammasome activation pathways. Kdo2 elicited stronger inflammatory responses in immortalized bone-marrow-derived macrophages (iBMDMs), as evidenced by robust TNF production, Caspase-11 cleavage, and IL-1α/IL-1β release. In contrast, IVa elicited minimal TNF secretion and failed to effectively induce non-canonical inflammasome activation. Global label-free quantitative proteomic analysis of iBMDMs stimulated with a low dose of immunogenic LPS displayed route-specific immune signatures: enrichment of TNF signaling, interferon-associated pathways, and mitochondrial metabolic remodeling. Equimolar amounts of low-acylated LPS failed to effectively induce these immune signatures, supporting a threshold-dependent model in which the lipid A structure and route of exposure define inflammatory progression. Collectively, our findings provide mechanistic insight into how lipid A structural variation modulates macrophage immune programming and cytosolic inflammasome activation.

## 1. Introduction

Macrophages are myeloid cells that act as a first line of defense against bacterial infections, including those caused by Gram-negative bacteria [1,2]. Lipopolysaccharide (LPS), a major component of the outer membrane of Gram-negative bacteria, acts as a potent immunomodulator in host cells [3,4]. Macrophages recognize pathogen-associated molecular patterns (PAMPs) using pattern recognition receptors (PRRs) and initiate downstream signaling cascades. Toll-like receptor 4 (TLR4) is a primary PRR used by immune cells to recognize LPS from Gram-negative bacteria [5,6,7]. TLR4 detects Gram-negative bacteria by sensing LPS, initiating inflammatory, antimicrobial, and interferon responses.

Systemic Gram-negative bacterial infections often result in LPS-driven septic shock [8,9], a critical and major cause of patient deaths worldwide. The LPS structure is composed of three parts: lipid A, containing two 3-deoxy-D-manno-octulosonic acid residues (Kdo2-lipid A); a core polysaccharide; and O-antigen repeats [10]. Kdo2-lipid A forms the membrane-embedded core of LPS and embodies the active stimulant for host-TLR4 signaling [11,12]. Modification of bacterial outer membrane components, such as lipid A, results in reduced recognition by host immune cells [13]. Therefore, deacylation of lipid A is a common strategy used by pathogenic microbes to evade host immune responses [14]. This enables bacterial growth without early immune recognition, leading to systemic dissemination, vascular escape, and ultimately bacteremia [15,16].

The phosphate groups and the length and number of fatty acyl chains in Kdo2-lipid A play critical roles in TLR4 activation [17,18,19]. A classic example, *Yersinia pestis,* is primarily transmitted through the bites of infected fleas. At 21–27 °C, *Y. pestis* synthesizes hexa-acylated lipid A. At body temperature, *Y. pestis* shifts to tetra-acylated lipid A, which reduces immune recognition and allows bacterial replication during early infection [11,14]. Similarly, *Helicobacter pylori* modifies lipid A acylation to produce weaker TLR4 stimulation [6,20].

Beyond TLR4-dependent recognition of extracellular LPS, LPS can access the cytosol through multiple routes, including CD14 (mCD14)-mediated internalization and delivery via outer membrane vesicles (OMVs) produced by Gram-negative bacteria, collectively enabling activation of a TLR4-independent surveillance pathway mediated by Caspase-11 [21,22]. Cytosolic LPS directly activates Caspase-11, resulting in gasdermin D pore formation. This process, known as non-canonical inflammasome activation, leads to canonical inflammasome–independent IL-1α release [21,23]. In contrast, IL-1β secretion requires secondary activation of the NLRP3 inflammasome and Caspase-1 downstream of gasdermin D–mediated potassium efflux [24]. This response eliminates intracellular replication niches, promotes inflammatory cytokine release, and exposes bacteria to extracellular immune clearance, thereby contributing to host defense against invasive Gram-negative pathogens [25].

Structural variation in lipid A acylation, including hexa-acylated and tetra-acylated forms, critically influences host immune recognition of Gram-negative bacteria through TLR4-dependent and -independent pathways and non-canonical inflammasome activation [14,26,27]. However, mechanisms by which bacteria evade early immune detection remain poorly defined, particularly under conditions of low-level immune activation during initial infection, where system-level proteomic remodeling remains largely unexplored. To address this, we examined global proteomic changes induced by low-dose Kdo2 (Kdo_2_-lipidA) and IVa (Lipid IVa) across TLR4-dependent and -independent and non-canonical inflammasome pathways to define how immune cells differentially respond to low doses of strong versus weak lipid A agonists and how this shapes TLR4-dependent and -independent innate immune signaling programs, including non-canonical inflammasome activation.

## 2. Materials and Methods

### 2.1. Generation and Culture of Immortalized Bone-Marrow-Derived Macrophages

Immortalized bone-marrow-derived macrophages (iBMDMs) were generated from WT mice using a previously described protocol [28]. Briefly, bone marrow progenitor cells were isolated from both mouse strains and differentiated into macrophages in complete DMEM (cDMEM) containing 25 ng/mL M-CSF, supplemented with 10% heat-inactivated fetal bovine serum and 5% horse serum. Cells were cultured in 10 cm dishes for 3 days. On day 4, 3 mL of fresh differentiation media was added, and the cultures were maintained until day 7. Bone-marrow-derived macrophages were then immortalized by replacing half of the differentiation media with 0.45 µm MCE-filtered J2-cell supernatant. The concentration of M-CSF was gradually eliminated, yielding M-CSF–independent immortal macrophages.

### 2.2. U937-Derived Macrophages

U937 (CRL1593.2) cells were cultured in complete RPMI (10% HI-FBS and 2 mM Glutamine) at a density below 2 × 10^6^ cells/mL. Cells were plated in fresh medium with 20 ng/mL during macrophage differentiation for 48 h prior to LPS stimulation or transfection.

### 2.3. Cell Treatment and Stimulation

iBMDMs were cultured under pathogen-free conditions in DMEM supplemented with 10% heat-inactivated FBS. For extracellular stimulation, cells were treated with the indicated concentrations of Kdo2 or lipid IVa (kindly provided by Prof. Robert K. Ernst, University of Maryland, Baltimore), with *E. coli* LPS (Sigma-Aldrich) used as a positive control in selected experiments. For intracellular stimulation, iBMDMs were transfected with the indicated concentrations of Kdo2 or lipid IVa. Lipids were complexed with Mirus TKO transfection reagent (Mirus) and incubated for 20 min prior to addition to the cells, followed by stimulation for 24 h. For double activation (non-canonical inflammasome activation), macrophages were primed with Pam3CSK4 (P3C) (InvivoGen) for 6 h prior to transfection with Kdo2 or lipid IVa for an additional 24 h.

### 2.4. Real-Time PCR

The RNeasy kit (QIAGEN) was used for RNA isolation from cell culture. Total RNA (1 μg) was converted to cDNA using a High-Capacity cDNA Reverse Transcription Kit (Thermo Scientific, Waltham, MA, USA), and real-time PCR was performed using the Power SYBR Green PCR Master Mix (Thermo Scientific) with specific primers to determine gene expression level. The primers are listed in Supplementary Table S1. The ^ΔΔCt^ method was used for determining relative gene expression, and beta-actin was used as a housekeeping gene.

### 2.5. Western Blot Analysis

iBMDMs were lysed in Pierce RIPA buffer (Thermo Fisher Scientific) supplemented with Halt protease and phosphatase inhibitor cocktails (Thermo Fisher Scientific). Total protein concentration was determined using a BCA protein assay (Thermo Fisher Scientific). Equal amounts of protein (20 µg) were resolved on NuPAGE 4–12% Bis-Tris polyacrylamide gels (Invitrogen) at 200 V for 1 h and transferred onto PVDF membranes (Thermo Fisher Scientific). Membranes were blocked in 5% BSA for 1 h, followed by incubation with primary antibodies overnight at 4 °C and secondary antibodies for 1 h at room temperature. Antibodies used are listed in Supplementary Table S2. Protein bands were detected using ECL substrate (Thermo Fisher Scientific) and visualized with a ChemiDoc imaging system (Bio-Rad). Densitometric analysis was performed using ImageJ Fiji 2.17.0.

### 2.6. Proteomic Analysis

*Sample Preparation*: Protein samples containing 1× protease inhibitor (20 μg) were prepared in 20 μL of 6 M urea in 50 mM triethylammonium bicarbonate (TEAB). Samples were reduced by 5 mM tris(2-carboxyethyl) phosphine (TCEP) for 30 min at 37 °C with shaking at 1000 rpm. Alkylation was performed by adding iodoacetamide (IAA) to a final concentration of 24 mM and incubating for 30 min at room temperature in the dark. Following alkylation, samples were immediately diluted with 50 mM TEAB to reduce the urea concentration to below 1 M. Proteins were digested overnight at 37 °C with sequencing-grade trypsin at a 1:25 (enzyme:protein) ratio with shaking at 1000 rpm. Digestion was terminated by adding trifluoroacetic acid (TFA) to a final concentration of 5% (*v*/*v*), facilitating cleavage of acid-labile detergents and precipitation of insoluble material. Samples were centrifuged at 12,000× *g* for 10 min at 4 °C, and the peptide-containing supernatant was transferred to clean tubes. Peptides were desalted using C18 ZipTips (Millipore) prior to LC–MS/MS analysis. *Mass Spectrometry*: An Orbitrap Fusion Eclipse with an EASY-Spray ion source (Thermo Fisher Scientific, San Jose, CA, USA) coupled to a Thermo UltiMate 3000 (Thermo Fisher Scientific) was used for LC-MS/MS experiments. A total of 1 μg of peptides was injected for LC-MS/MS analysis. Peptides were trapped on an Acclaim C18 PepMap 100 trap column (5 µm particles, 100 Å pores, 300 µm i.d. x 5 mm, Thermo Fisher Scientific) and separated on a PepMap RSLC C18 column (2 µm particles, 100 Å pores, 75 µm i.d. x 50 cm, Thermo Fisher Scientific) at 40 °C. The LC steps were: 98% mobile phase A (0.1% *v*/*v* formic acid in H2O) and 2% mobile phase B (0.1% *v*/*v* formic acid in ACN) from 0 to 5 min; 2% to 35% linear gradient of mobile phase B from 5 to 155 min; 35% to 85% linear gradient of mobile phase B from 155 to 157 min; 85% mobile phase B from 157 to 170 min; 85% to 2% linear gradient of mobile phase B from 170 to 172 min; 2% of mobile phase B from 172 to 190 min. Eluted peptides were ionized in positive-ion polarity at 2.1 kV of spraying voltage. MS1 full scans were recorded in the range of *m*/*z* 375 to 1500 with a resolution of 120,000 at 200 *m*/*z* using the Orbitrap mass analyzer. Automatic gain control and maximum injection time were set to standard and auto, respectively. The top 3sec data-dependent acquisition mode was used to maximize the number of MS2 spectra from each duty cycle. Higher-energy collision-induced dissociation (HCD) was used to fragment selected precursor ions with a normalized collision energy of 27. MS2 scans were recorded using an automatic scan range with a resolution of 15,000 at 200 *m*/*z* using the Orbitrap mass analyzer. Data analysis, label-free quantification, and statistical analysis were performed using Proteome Discoverer 3.1 with the Chimerys search engine (Thermo Fisher Scientific). Briefly, the raw files were searched against the mouse uniport database (download date, 15 March 2024) with the list of common protein contaminants. The groups and conditions were specified to obtain the quantification ratios, and adjusted *p*-values were calculated using the Benjamini–Hochberg method. The data visualization was performed using R packages. The differentially expressed proteins were identified using log2 fold change less than −0.585 (0.67-fold change) for downregulated proteins and log2 fold change more than 0.585 (1.5-fold change) for upregulated proteins, combined with the adjusted *p*-value less than 0.05. Volcano plots were generated by ggplot2. All the significant proteins were used as input to identify the significantly enriched GO and pathway analyses, and the bubble plots were generated by clusterProfiler [29,30,31,32]. KEGG pathway analyses were searched against the mouse KEGG [33] genome database, and the bubble charts were generated by pathfinder [34]. The adjusted *p*-value was calculated using Bonferroni’s method. The heatmap was generated by pheatmap. Protein–protein interaction (PPI) network analysis was performed using the STRING database (v12.0) with *Mus musculus* as the reference. A high-confidence interaction score threshold of 0.7 was applied to ensure reliable interactions. To identify functional modules within the network, k-means clustering was performed within STRING with the number of clusters set to k = 4. The resulting clusters were further analyzed for functional enrichment to determine their biological significance. Full gene nomenclature and corresponding protein identifiers are provided in Supplementary Table S3. The mass spectrometry proteomics data were deposited in the ProteomeXchange Consortium [35] via the PRIDE [36] partner repository with the dataset identifier PXD042097 (temporary reviewer account details: Username: reviewer_pxd075293@ebi.ac.uk Password: hsKSxG1W5D1s).

### 2.7. Statistical Analysis

Statistical analyses were performed using GraphPad Prism version 10.2.0 (GraphPad Software), Proteome Discoverer (Thermo Fisher Scientific), and the R statistical environment (version 4.5.2). Data are presented as mean ± standard deviation (SD), unless otherwise indicated. Statistical details, including the tests used and significance thresholds, are provided in the Section 2 and the corresponding figure legends.

## 3. Results

### 3.1. Lipid IVa, but Not E. coli LPS or Kdo2 Lipid A, Elicits Host Species-Specific Macrophage Responses

**Figure 1:**
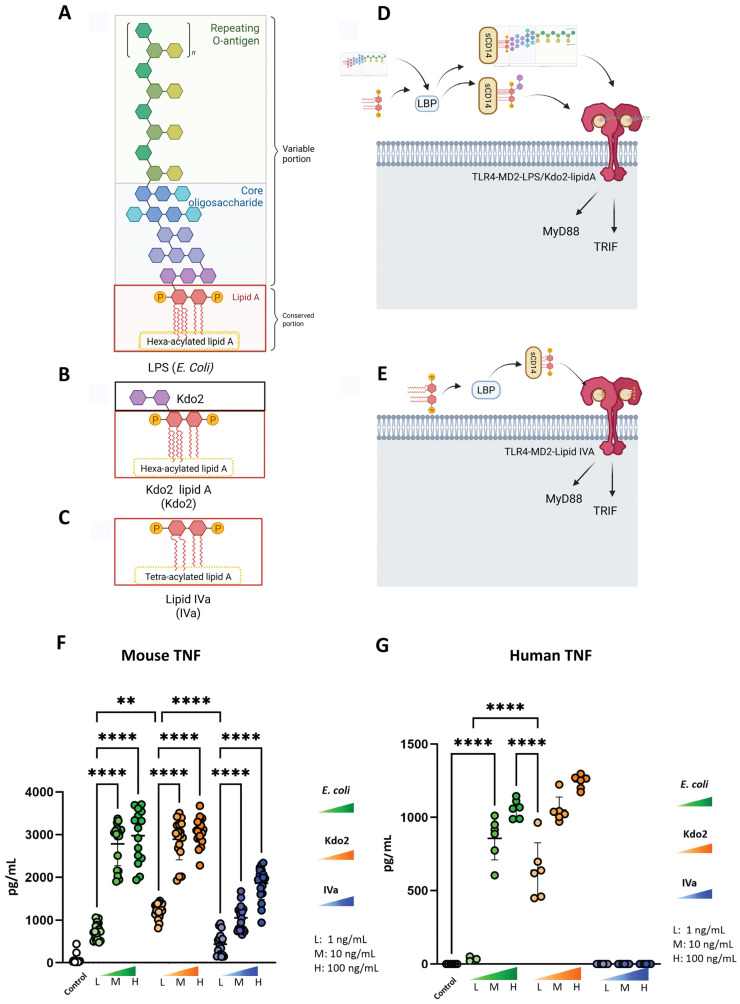
**Structural overview of LPS, Kdo2-lipid A, and lipid IVa, and cytokine responses to dose-dependent stimulation:** (**A**) Schematic representation of lipopolysaccharide (LPS) from *E. coli*, composed of repeating O-antigen, core oligosaccharide, and hexa-acylated lipid A. (**B**) Schematic representation of Kdo2-lipid A, consisting of two Kdo residues linked to hexa-acylated lipid A. (**C**) Schematic representation of lipid IVa, composed of a tetra-acylated lipid A backbone. (**D**) Schematic illustration of Kdo2-lipid A or LPS engagement with TLR4 signaling. LPS-binding protein (LBP) binds to hexa-acylated lipid A from either *E. coli* LPS or Kdo2-lipid A. These molecules are transferred to soluble CD14 (sCD14), which then delivers them to the MD2–TLR4 complex, initiating TLR4 signaling through the formation and dimerization of TLR4–MD2–LPS or TLR4–MD2–Kdo2 ternary complexes. (**E**) Schematic illustration of lipid IVa engagement with TLR4 signaling. LBP binds to tetra-acylated lipid A (lipid IVa), which is transferred to soluble CD14 (sCD14) and subsequently delivered to the MD2–TLR4 complex. This interaction leads to formation of the TLR4–MD2–lipid IVa complex, resulting in TLR4 activation. Illustrations were created in BioRender. Nita-Lazar, A. (2026) https://BioRender.com/vogoub2 (**F**) iBMDMs were treated with dose-dependent concentrations (1, 10, and 100 ng/mL) of *E. coli* LPS Kdo2 or IVa for 6 h. TNF levels were determined by ELISA. Data are representative of three independent experiments and are shown as mean ± SD: , ** *p* < 0.01, **** *p* < 0.0001 (one-way ANOVA). (**G**) U937-derived macrophages were treated with dose-dependent concentrations (1, 10, and 100 ng/mL) of *E. coli* LPS, Kdo2, or IVa for 6 h. TNF levels were determined by ELISA. Data are representative of three independent experiments and are shown as mean ± SD: **** *p* < 0.0001 (one-way ANOVA followed by Tukey’s multiple comparisons test).

To facilitate comparison of lipid A structural features, including their acylation state and their engagement with TLR4 signaling, schematic illustrations of LPS, Kdo2, and IVa, as well as their interaction with TLR4, are shown in Figure 1A–E. To determine the dose-dependent inflammatory responses induced by Kdo2 and IVa in macrophages, we performed dose–response experiments using high (H, 100 ng/mL), medium (M, 10 ng/mL), and low (L, 1 ng/mL) concentrations of Kdo2, lipid IVa, and *E. coli* LPS as a positive control. iBMDMs and U937-derived human macrophages were stimulated, and TNF secretion was measured. All stimuli induced dose-dependent TNF secretion in mouse macrophages (Figure 1A). In human macrophages, *E. coli* LPS and Kdo2 elicited comparable TNF responses (Figure 1B). In contrast, IVa induced a weaker TNF response in mouse macrophages compared with Kdo2 and *E. coli* LPS. Notably, IVa failed to induce TNF secretion at any tested dose in human macrophages (Figure 1B), indicating species-specific differences in macrophage activation by lipid IVa. Because both Kdo2 and IVa showed dose-dependent responses in mouse macrophages, we selected 10 ng/mL as a high-dose condition that produced robust TNF secretion and 1 ng/mL as a low-dose condition for subsequent experiments.

### 3.2. Kdo2 Induces Stronger M1-Associated Macrophage Activation than Lipid IVa

**Figure 2:**
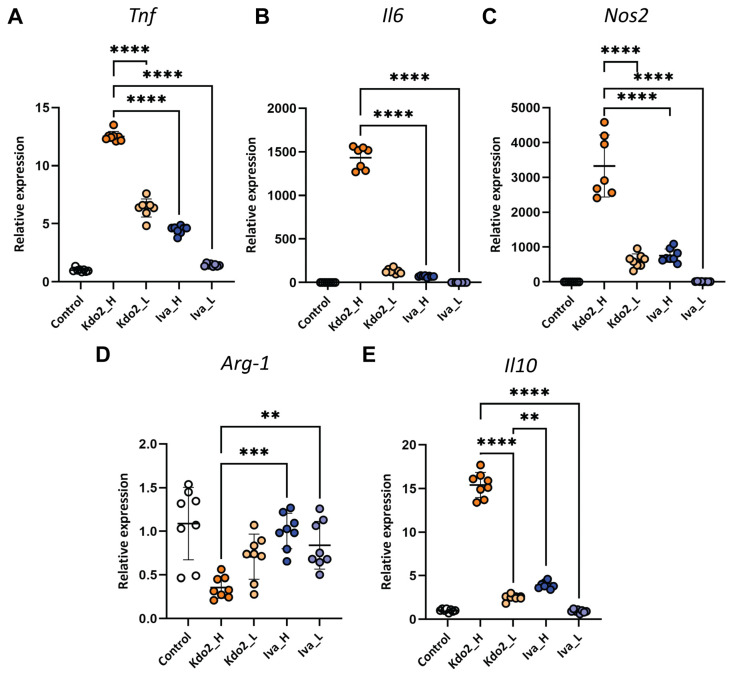
Gene expression profile of dose-dependent Kdo2 and IVa stimulation: (**A**–**E**) iBMDMs were treated with high and low doses of Kdo2 or IVa for 6 h (Kdo2_H, Kdo2L, IVa_H, and IVa_L). Relative gene expression of *Il10*, *Arg-1*, *Il6*, *Tnf*, and *iNOS* was determined using real-time PCR. Beta-actin was used as an internal control. Data are representative of 3 independent experiments and shown as mean ± SD: ** *p* < 0.01, *** *p* < 0.001, **** *p* < 0.0001 (one-way ANOVA followed by Tukey’s multiple comparisons test).

The balance of macrophage polarization between M1 (pro-inflammatory) and M2 (anti-inflammatory) states plays an important role in macrophage biology. To characterize macrophage polarization following Kdo2 and IVa treatment, iBMDMs were stimulated with a high dose (10 ng/mL) and low dose (1 ng/mL) of each ligand. Real-time PCR analysis revealed that Kdo2 induced dose-dependent upregulation of the M1-associated genes *Il6*, *Tnf*, and *Nos2* (*iNOS*) (Figure 2A–C), indicating a strong pro-inflammatory activation profile. Meanwhile, IVa exhibited a similar trend but a markedly weaker induction of these pro-inflammatory markers compared with Kdo_2_ lipid A. In contrast, both Kdo2 and IVa reduced expression of the M2-associated marker *Arg1* (Figure 2D) relative to unstimulated cells, while *Il10* expression (Figure 2E) was selectively increased by high-dose Kdo2 in a dose-dependent manner. Together, these data indicate that both Kdo2 and IVa promote M1-associated macrophage polarization, with Kdo2 eliciting a more dominant response.

### 3.3. Kdo2 Elicits Stronger Caspase-11 Activation and Non-Canonical Inflammasome Activation

**Figure 3:**
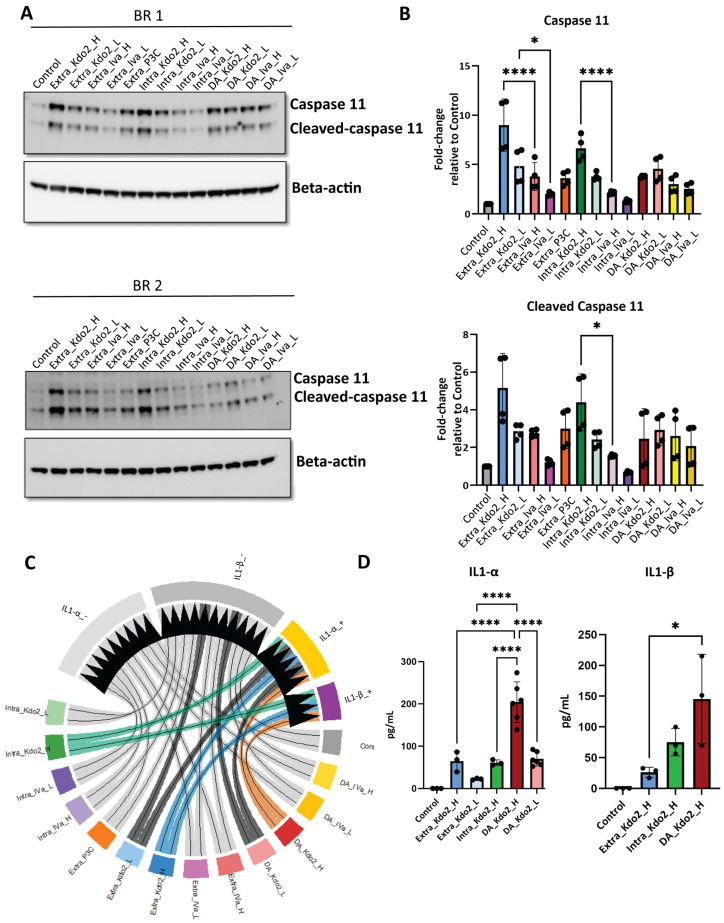
Characterization of non-canonical inflammasome activation in macrophages: (**A**) iBMDMs were stimulated with Kdo2 or lipid IVa via extracellular, intracellular, or double-activation (non-canonical inflammasome activation) routes at high (H) and low (L) doses. Extracellular treatments are denoted as Extra_Kdo2_H, Extra_Kdo2_L, Extra_IVa_H, and Extra_IVa_L; intracellular treatments as Intra_Kdo2_H, Intra_Kdo2_L, Intra_IVa_H, and Intra_IVa_L; and double-activation treatments as DA_Kdo2_H, DA_Kdo2_L, DA_IVa_H, and DA_IVa_L. Cell lysates were collected and analyzed by Western blot for Caspase-11, with β-actin as a loading control. Data are representative of two independent experiments. (**B**) Densitometry analysis using image J. Data are representative of 2 independent experiments. Each blot was analyzed twice for accuracy. Data are representative of 2 independent experiments and shown as mean ± SD: * *p* < 0.05, , **** *p* < 0.0001 (one-way ANOVA followed by Tukey’s multiple comparisons test). (**C**) IL1-α and IL1-β release in the supernatants of all the conditions was measured by ELISA. The chord diagram was generated to summarize the release of IL1-α and IL1-β. (**D**) IL1-α and IL1-β release in the supernatants of all the conditions was measured by ELISA. Data are representative of 3 independent experiments and shown as mean ± SD: * *p* < 0.05, **** *p* < 0.0001 (one-way ANOVA).

Caspase-11 is a key sensor of cytoplasmic LPS and a central mediator of non-canonical inflammasome activation. To investigate Caspase-11 activation in response to Kdo2 and lipid IVa, we examined three experimental conditions: (i) extracellular stimulation to assess TLR4-dependent activation (Extra_Kdo2/Extra_IVa); (ii) direct cytosolic delivery by transfection to evaluate TLR4-independent sensing (Intra_Kdo2/Intra_IVa); and (iii) double activation consisting of priming with Pam3CSK4 (P3C) for 6 h followed by transfection with either Kdo2 or IVa for 24 h to induce non-canonical inflammasome activation (DA_Kdo2/DA_IVa). We also use extracellular P3C stimulation for 6 h for another control of priming. The Caspase-11 activation was assessed by measuring total Caspase-11 and cleaved Caspase-11, as well as IL-1α and IL-1β release as readouts of cytosolic sensing and non-canonical inflammasome activation. Both Caspase-11 and cleaved Caspase-11 were detectable across all stimulation conditions and doses compared with unstimulated controls, with Kdo2 consistently inducing more robust Caspase-11 cleavage than IVa. Caspase-11 activation was further enhanced under high-dose Kdo2 stimulation. In contrast, IVa induced weaker activation of Caspase-11 and cleaved Caspase-11 across conditions, although responses remained significantly elevated relative to unstimulated cells (Figure 3A,B). Given that Caspase-11 autolysis is required for non-canonical inflammasome activation and IL-1α release, whereas IL-1β release requires secondary activation of the canonical inflammasome, we next examined cytokine release. IL-1α release was observed across all stimulation conditions except low-dose transfection with Kdo2, with high-dose Kdo2 inducing the strongest response. In contrast, IL-1α release following IVa stimulation was detected only under double-activation conditions. A similar pattern was observed for IL-1β release, which was detected only under double-activation conditions with Kdo2, consistent with a requirement for secondary inflammasome activation (Figure 3C,D). These data indicate that IVa does not induce detectable IL-1β release under the conditions tested, consistent with a lack of secondary canonical inflammasome activation. Given the robust responses observed under high-dose stimulation, we next selected low-dose lipid A stimulation to better reflect physiological conditions in which immune cells encounter limited bacterial burden during early stages of infection, allowing us to assess how reduced acylation of lipid A impacts macrophage defense mechanisms.

### 3.4. TLR4 and Caspase-11–Dependent Activation Using Low-Dose Kdo2 and IVa Results in Distinct Proteomic Profiles

**Figure 4:**
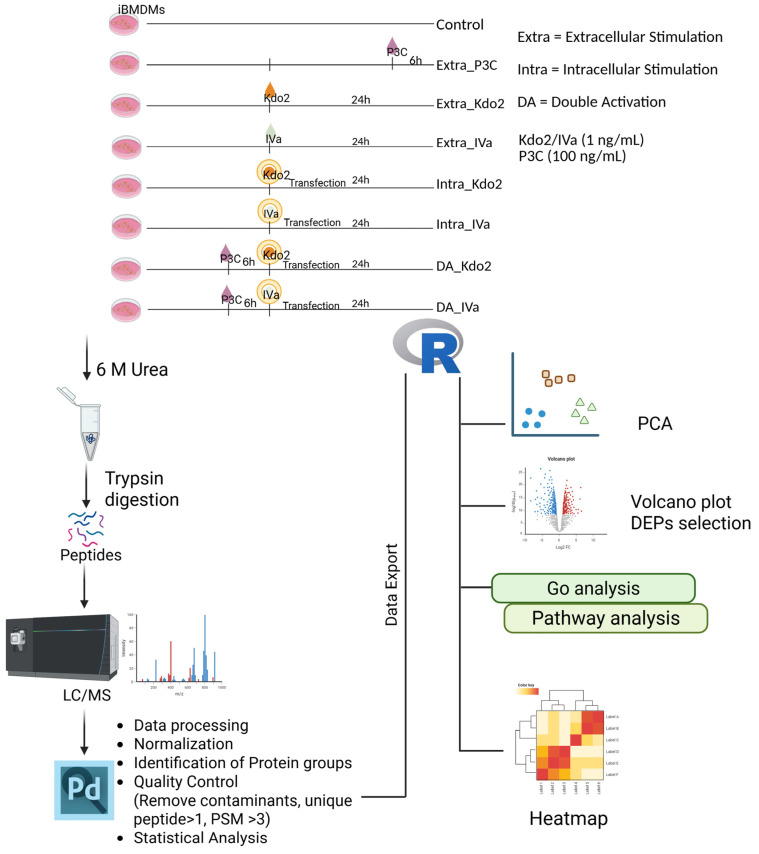
Schematic overview of the experimental workflow for proteomics. iBMDMs were treated with indicated conditions. All the cell lysates were collected at the same time to make sure that cells were the same age. Cells were lysed, and the protein samples were processed with sample preparation for mass spectrometry analysis. Following the analysis, mass spectrometry raw files were analyzed using Proteome Discoverer 3.1, then the analysis results were exported and the DEP selection, volcano plot, GO analysis, and pathway analysis were performed using R packages. Created in BioRender. Nita-Lazar, A. (2026) https://BioRender.com/k46grl5.

To assess global proteomic changes in macrophages in response to distinct lipid A species under extracellular and intracellular stimulation, as well as non-canonical inflammasome activation, we performed quantitative proteomic analyses as outlined in the study design (Figure 4). Principal component analysis (PCA) revealed clear separation among experimental conditions, indicating distinct proteomic profiles in response to the indicated stimulations (Figure S2). Pairwise correlation analysis demonstrated high reproducibility among biological replicates, supporting the robustness of the dataset (Figure S1A). Consistently, unsupervised heatmap analysis showed distinct proteomic patterns across all conditions (Figure S1B), with similar clustering patterns observed among biological replicates within each condition. Overall, a total of 4872 proteins were identified across all conditions. To gain insight into the underlying biological functions, we identified differentially expressed proteins (DEPs) between the control and each stimulation condition. Robust numbers of DEPs were detected across different comparisons and stimuli (Figure S3). These data suggest that both lipid A structural differences and routes of stimulation influence global proteomic profiles.

### 3.5. Low-Dose Extracellular Stimulation with Kdo2 and IVa Elicits Distinct TLR4-Associated Proteomic Signatures

**Figure 5:**
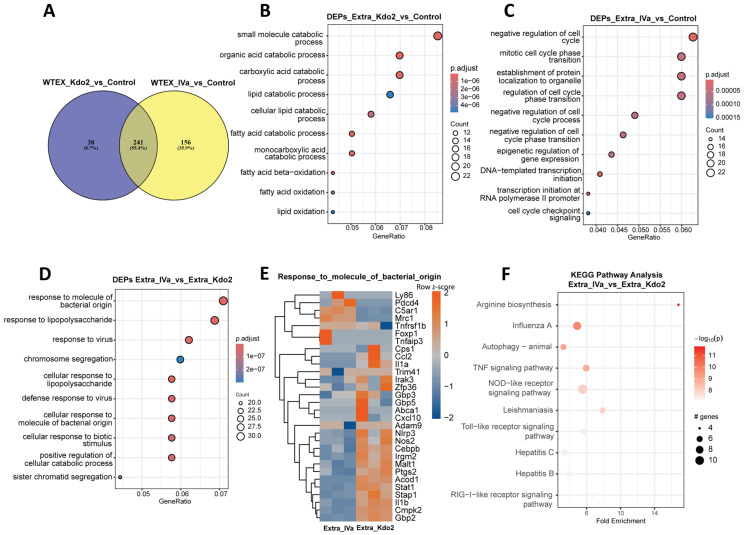
Proteomic profiling of macrophages following extracellular stimulation with Kdo2 and lipid IVa: (**A**) Venn diagram illustrates shared and unique DEPs identified after extracellular stimulation with Kdo2 and IVa as compared to unstimulated control. (**B**) Gene Ontology (GO) biological process enrichment analysis of unique DEPs identified after extracellular stimulation with Kdo2 compared to unstimulated control. Enrichment analysis was performed using clusterProfiler and visualized as bubble plot. The *x*-axis represents fold enrichment, and the *y*-axis lists enriched GO terms. Bubble size indicates the number of DEPs associated with each GO term, while color intensity represents −log10 (*p* value), with darker red indicating stronger enrichment significance. (**C**) Gene Ontology (GO) biological process enrichment analysis of unique DEPs identified after extracellular stimulation with IVa compared to unstimulated control. Enrichment analysis was performed using clusterProfiler and visualized as bubble plot. The *x*-axis represents fold enrichment, and the *y*-axis lists enriched GO terms. Bubble size indicates the number of DEPs associated with each GO term, while color intensity represents −log10 (*p* value), with darker red indicating stronger enrichment significance. (**D**) GO biological process enrichment analysis of all DEPs identified under extracellular stimulation with Kdo2 and IVa comparison. Results are displayed as a bubble plot with gene ratio on the *x*-axis and enriched GO terms on the *y*-axis. Bubble size represents the number of DEPs per GO term, and color intensity reflects −log10 (*p* value).(**E**) Heatmap illustrating DEPs involved in the response to molecules of bacterial origin following extracellular stimulation with Kdo2 and IVa. (**F**) KEGG pathway enrichment analysis of DEPs identified after extracellular stimulation with Kdo2 and IVa. The x-axis represents fold enrichment and the y-axis lists enriched pathways. Bubble size indicates the number of DEPs associated with each pathway, and color intensity corresponds to −log10(*p* value), with darker red indicating higher statistical significance.

To translate the observed proteomic remodeling into functional insights, we first generated a Venn diagram of differentially expressed proteins (DEPs) identified in Kdo2- and IVa-treated cells compared with controls. We found that 241 DEPs were shared between the two conditions, whereas IVa exhibited a distinct set of uniquely regulated proteins, indicating divergent proteomic profiles (Figure 5A). Moreover, we subjected the differentially expressed proteins from each stimulation condition to biological process (BP) and pathway enrichment analyses. We first examined extracellular stimulation with lipid Kdo2 and IVa at 24 h. BP enrichment analysis revealed that lipid Kdo2 stimulation was primarily associated with processes related to fatty acid metabolism and fatty acid oxidation (Figure 5B). In contrast, extracellular stimulation with IVa showed distinct biological processes, with the top enriched terms associated with negative regulation of the cell cycle, cell cycle checkpoint signaling, and epigenetic regulation of gene expression (Figure 5C). These findings highlight divergent biological responses induced by lipid Kdo2 and IVa under extracellular stimulation. To further characterize these differences, we performed BP enrichment analysis using differentially expressed proteins from lipid Kdo2 and IVa stimulation. Enriched biological processes included responses to molecules of bacterial origin, lipopolysaccharide, and viruses (Figure 5D). Under extracellular stimulation, Kdo2 induced increased abundance of proteins associated with inflammatory signaling (NOS2, PTGS2, CEBPB, and MALT), interferon-associated responses (STAT1, GBP2, CMPK2, and IRGM2), inflammasome priming (NLRP3 and IL1β), and immunometabolic regulation (ACOD1) (Figure 5E). Collectively, these responses were not equivalently observed following IVa stimulation, indicating that extracellular Kdo2 elicits a robust surface TLR4-dependent proteomic program. These findings were further supported by pathway analysis, which identified enrichment of Toll-like receptor signaling, and RIG-I–like receptor signaling pathways (Figure 5F). Taken together, these results suggest that low-dose extracellular stimulation with lipid Kdo2 and IVa differentially reprogram the macrophage proteome.

### 3.6. Low-Dose Intracellular Delivery of Kdo2 and IVa Elicits Distinct Cytosolic Proteomic Signatures

**Figure 6:**
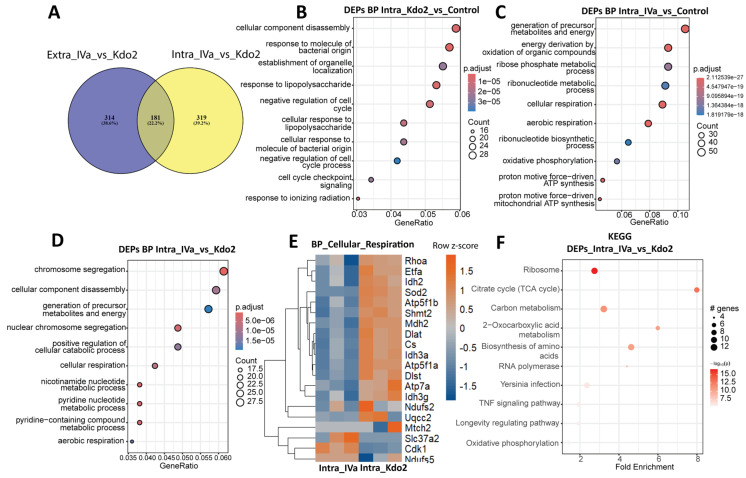
Proteomic profiling of macrophages following intracellular stimulation with Kdo2 and IVa: (**A**) Venn diagram showing shared and unique DEPs identified following extracellular or intracellular stimulation with Kdo2 and IVa. (**B**,**C**) Gene Ontology (GO) biological process enrichment analysis of unique DEPs identified after intracellular stimulation with Kdo2 and IVa. Enrichment analysis was performed using clusterProfiler and visualized as bubble plots. The *x*-axis represents fold enrichment, and the *y*-axis lists enriched GO terms. Bubble size indicates the number of DEPs associated with each GO term, while color intensity represents −log10 (*p* value), with darker red indicating stronger enrichment significance. (**D**) GO biological process enrichment analysis of all DEPs identified under extracellular and intracellular stimulation with Kdo2 and IVa. Results are displayed as a bubble plot with fold enrichment on the *x*-axis and enriched GO terms on the *y*-axis. Bubble size represents the number of DEPs per GO term, and color intensity reflects −log10 (*p* value). (**E**) Heatmap illustrating DEPs involved in cellular respiration following intracellular stimulation with Kdo2 and IVa. (**F**) KEGG pathway enrichment analysis of DEPs identified under intracellular stimulation with Kdo2 and IVa. The *x*-axis represents fold enrichment, and the *y*-axis lists enriched pathways. Bubble size indicates the number of DEPs associated with each pathway, and color intensity corresponds to −log10 (*p* value), with darker red indicating higher statistical significance.

To further investigate the impact of intracellular lipid A sensing on macrophage proteomes, we directly transfected Kdo2 and IVa and compared the resulting differentially expressed proteins (DEPs) with those identified under extracellular stimulation. Comparison of DEPs across routes revealed 181 proteins shared between extracellular and intracellular stimulation, whereas 314 and 319 DEPs were uniquely enriched under extracellular and intracellular conditions, respectively (Figure 6A). These findings indicate substantial route-dependent proteomic remodeling. We next performed biological process (BP) enrichment analysis for intracellular Kdo2 and IVa stimulation relative to controls. Intracellular Kdo2 transfection enriched BPs reflective of LPS-associated immune responses, including cellular component disassembly, response to molecules of bacterial origin, and cellular response to lipopolysaccharide (Figure 6B). In contrast, intracellular IVa transfection preferentially enriches metabolic processes, including the generation of precursor metabolites and energy, cellular respiration, and oxidative phosphorylation (Figure 6C). Direct comparison of Kdo2 and IVa under intracellular conditions revealed the top enriched BPs associated with cellular respiration, pyridine nucleotide metabolism, and aerobic respiration (Figure 6D). Given the central role of mitochondrial respiration in immune activation, we further examined proteins associated with these BPs. Following intracellular delivery of Kdo2, proteins involved in mitochondrial bioenergetic pathways—including TCA cycle enzymes (CS, MDH2, IDH2/IDH3A/IDH3G, and DLAT), oxidative phosphorylation components (ATP5F1B), electron transfer proteins (ETFA), and redox regulators (SOD2)—were increased, consistent with mitochondrial metabolic remodeling (Figure 6E). Notably, these proteins were more prominently expressed in Kdo2-treated cells compared with IVa. These findings were further supported by the KEGG pathway analysis, which identified enrichment of ribosome, TNF signaling, and oxidative phosphorylation pathways (Figure 6F). Taken together, Kdo2 and IVa exhibit stimulus- and route-specific proteomic landscapes.

### 3.7. Differences in Non-Canonical Inflammasome Activation by Low-Dose Kdo2 and IVa Correlate with Distinct Proteomic Landscapes

**Figure 7:**
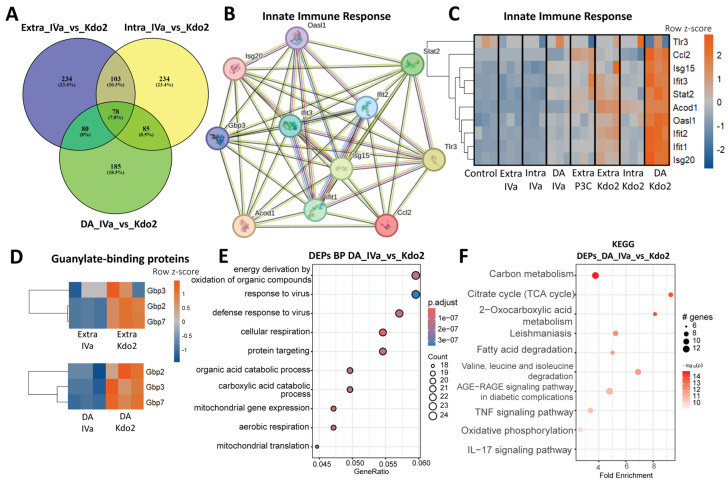
Proteomic profiling of macrophages following non-canonical inflammasome activation with Kdo2 and IVa: (**A**) Venn diagram showing shared and unique DEPs identified following extracellular or intracellular stimulation and double activation with Kdo2 and IVa. (**B**) Protein–protein interaction (PPI) network of DEPs was constructed via STRING database. The network focused on innate immune responses contains 11 nodes and 49 edges, with an average node degree of 8.91 and clustering coefficient of 0.898. Each node corresponds to a protein, while edges denote predicted or experimentally supported protein–protein associations. Colored nodes indicate query proteins and their first shell of interactors, while white nodes represent the second shell of interactors. Filled nodes indicate proteins with known or predicted three-dimensional structures, whereas empty nodes represent proteins with unknown structures. Edge colors denote different types of interaction evidence: light blue for curated database interactions; pink for experimentally determined interactions; green for gene neighborhood associations; red for gene fusion events; dark blue for gene co-occurrence; yellow for text-mining evidence; black for co-expression; and purple for protein homology. (**C**) Heatmap illustrates unique DEPs involved in innate immune response following 3 routes of stimulation with Kdo2, IVa, and P3C. (**D**) Heatmap illustrating unique differentially expressed proteins involved in GBP family upon extracellular stimulation and non-canonical inflammasome activation with Kdo2, IVa. (**E**) GO biological process enrichment analysis of all DEPs identified under non-canonical activation with Kdo_2_-lipid A and lipid IVa. Results are displayed as a bubble plot with fold enrichment on the *x*-axis and enriched GO terms on the *y*-axis. Bubble size represents the number of DEPs per GO term, and color intensity reflects −log10 (*p* value). (**F**) KEGG pathway enrichment analysis of identified under non-canonical activation with Kdo_2_-lipid A and lipid IVa. The *x*-axis represents fold enrichment, and the *y*-axis lists enriched pathways. Bubble size indicates the number of DEPs associated with each pathway, and color intensity corresponds to −log10 (*p* value), with darker red indicating higher statistical significance.

To further characterize the proteomic landscape of low-dose responses to Kdo2 and lipid IVa, we generated Venn diagrams of differentially expressed proteins (DEPs) comparing Kdo2 and IVa across three routes of stimulation. We identified 78 DEPs that were commonly and significantly regulated across all routes. To investigate functional relationships among these shared proteins, we performed cluster enrichment analysis using the STRING database. Network analysis revealed three major clusters, among which cluster 2 (11 proteins) was enriched for innate immune response, which is composed of antiviral activity and response to interferon-β, suggesting a conserved macrophage immune response across stimulation routes (Figure 7B). We next visualized the expression of these proteins using a heatmap and observed that interferon-associated proteins were more strongly upregulated following Kdo2 stimulation compared with IVa (Figure 7C). Notably, guanylate-binding proteins (GBPs), the interferon-inducible GTPases implicated in cell-autonomous defense against intracellular pathogens, were increased in both extracellular stimulation and non-canonical inflammasome activation following Kdo2 relative to IVa, indicating that low-dose Kdo2 is sufficient to elicit robust macrophage immune programs (Figure 7D). In addition, we examined proteins uniquely altered under non-canonical inflammasome activation. Proteins enriched in this condition were predominantly associated with mitochondrial bioenergetic pathways, including TCA cycle enzymes, fatty acid β-oxidation components, electron transport chain subunits, and mitochondrial ribosomal proteins, consistent with mitochondrial oxidative metabolic remodeling (Figure 7F). These findings suggest that mitochondrial metabolic remodeling may accompany and potentially modulate inflammasome-associated responses (Figure 7E). To further support this observation, we performed pathway enrichment analysis comparing double activation with IVa and Kdo2. The top enriched biological processes were associated with antiviral defense and innate immune responses. In addition, enriched pathways included the TCA cycle, fatty acid degradation, and oxidative phosphorylation (Figure 7F). Collectively, these results indicate that non-canonical stimulation induces coordinated metabolic and immune remodeling.

## 4. Discussion

Structural modification of lipid A plays a critical role in regulating host innate immune responses [37,38]. Hexa-acylated lipid A induces robust immune activation [39], whereas tetra-acylated lipid A functions as a weak agonist in murine macrophages due to incomplete activation of MyD88-independent signaling [40], and acts as an antagonist in human macrophages [41,42,43,44]. Structural variation in lipid A frequently arises through bacterial enzymatic modification and represents a strategy to modulate or evade host immune detection [14,45,46]. Consistent with these reports, our data demonstrate that Kdo2 acts as a strong agonist in macrophages, inducing robust TNF expression, whereas IVa exhibits markedly weaker stimulatory capacity in iBMDMs.

Macrophage polarization is strongly influenced by the microenvironment and context of stimulation, leading to distinct inflammatory (M1-like) or anti-inflammatory (M2-like) phenotypes [47]. In our study, both high and low doses of Kdo2 and IVa induced pro-inflammatory markers, including IL-6, TNF, and iNOS, while the canonical M2 marker was reduced relative to unstimulated controls. Although IL-10 was upregulated following stimulation with both ligands, this likely reflects compensatory negative feedback rather than induction of an M2 phenotype [48]. Together, these findings indicate that both Kdo2 and IVa promote M1-like polarization, differing primarily in the magnitude of inflammatory activation rather than polarization direction in iBMDMs.

Caspase-11 (in mice; Caspase-4/5 in humans) plays a central role in cytosolic LPS sensing and non-canonical inflammasome activation [49,50]. Upon cytosolic recognition of lipid A, Caspase-11 becomes activated and induces pyroptosis through cleavage of Gasdermin D (GSDMD), resulting in pore formation and the release of inflammatory mediators [49,51]. In this study, we evaluated three routes of Kdo2 and IVa stimulation: (i) traditional extracellular stimulation; (ii) cytosolic delivery via transfection; and (iii) double activation (DA), which facilitates non-canonical inflammasome activation.

We observed robust Caspase-11 cleavage following high-dose Kdo2 stimulation, whereas IVa induced minimal activation, consistent with reduced immunogenic potency. Notably, extracellular Kdo2 stimulation also activated Caspase-11, potentially via mCD14-mediated internalization of lipid A into the cytosol [21,52]. Intracellular delivery triggered Caspase-11 activation as well, though at lower levels compared to extracellular stimulation, with similar trends observed under DA conditions.

Together, these data support a threshold-dependent model of non-canonical inflammasome progression in which lipid A acylation state determines whether cytosolic sensing advances from GSDMD-mediated membrane permeabilization to secondary canonical inflammasome amplification. Highly acylated Kdo2 at high doses is sufficient to drive this transition, whereas tetra-acylated IVa fails to reach the signaling intensity required for IL-1 cytokine release. This distinction underscores how structural modification of lipid A may regulate the balance between inflammatory priming and full inflammasome execution.

Collectively, our findings support a threshold model in which lipid A acylation state and route of cellular entry cooperatively determine the magnitude and progression of cytosolic inflammasome activation. While both Kdo2 and IVa induce M1-like inflammatory priming, only highly acylated Kdo2 surpasses the activation threshold required for robust Caspase-11 cleavage and secondary canonical inflammasome amplification.

To model early immune recognition and potential immune evasion, we performed global proteomic profiling under low-dose Kdo2 and IVa stimulation across three activation routes. Under these subthreshold conditions, macrophages exhibited route-specific immune signatures, including interferon-associated programs and mitochondrial metabolic remodeling, without overt canonical inflammasome activation. Extracellular stimulation was enriched for Toll-like receptor signaling pathways, whereas intracellular stimulation preferentially engaged metabolic and respiratory pathways, highlighting route-specific immune–metabolic programming. Double stimulation with Kdo2 further enriches interferon-inducible proteins and guanylate-binding proteins (GBPs), key mediators of intracellular pathogen defense. For example, GBP2, GBP3, and GBP7, which we found, clearly upregulate in extracellular and double activation, and this set of proteins has been reported to be involved in non-canonical inflammasome activation [51,53,54,55,56].

**Figure 8:**
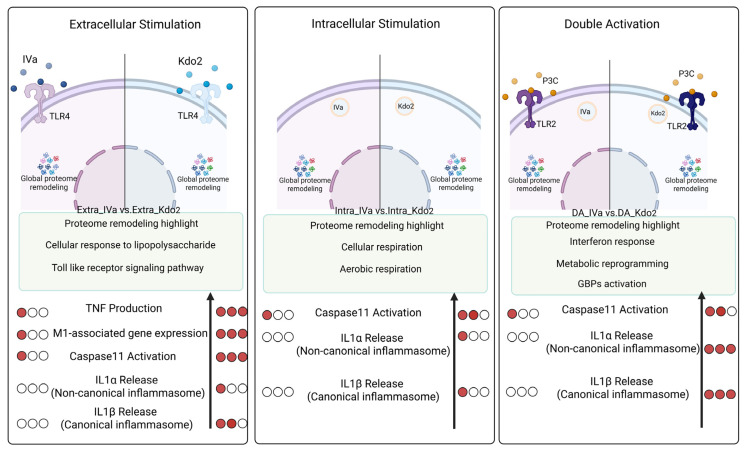
Proposed working model of route-dependent macrophage responses to Kdo2 and IVa: Schematic summary of how lipid A acylation and route of stimulation shape macrophage responses and proteome remodeling. (**Left**) Extracellular stimulation: Hexa-acylated Kdo2 induces robust TLR4 signaling, leading to strong TNF production, M1-associated gene expression, and Caspase-11 activation. In contrast, IVa elicits weaker activation. Proteomic analysis highlights enrichment of pathways related to cellular response to lipopolysaccharide and Toll-like receptor signaling. (**Middle**) Intracellular stimulation: Cytosolic delivery by transfection of lipid A activates Caspase-11–dependent pathways. Kdo2 induces stronger Caspase-11 activation and IL-1α release (non-canonical inflammasome), whereas lipid IVa shows attenuated responses. Proteome remodeling is associated with cellular and aerobic respiration pathways. (**Right**) Double activation (P3C priming + intracellular activation): Combined stimulation enhances inflammatory and interferon-related responses. Kdo2 induces stronger Caspase-11 activation and cytokine release, GBP activation, and interferon responses, whereas lipid IVa triggers lower interferon responses, metabolic reprogramming. Proteomic analysis highlights pathways associated with interferon signaling and immune–metabolic regulation. Dot intensity represents relative magnitude of response. This model summarizes findings from the present study. Illustrations were created in BioRender. Nita-Lazar, A. (2026) https://BioRender.com/mt4cf1m. The number of red circles indicates the strength of the response.

Together, these data suggest that lipid A acylation not only determines inflammatory potency but also shapes the trajectory of macrophage immune programming under low-dose conditions. Reduced acylation from six to four acyl chains may allow pathogens to remain below the activation threshold required for full inflammasome amplification while still engaging partial immune signaling. Our global proteomic analysis provides mechanistic insight into how lipid A structural variation and subcellular route of exposure coordinate macrophage immune responses. These findings are summarized in Figure 8. Further functional studies will be required to dissect the precise contribution of individual proteomic modules to inflammasome regulation and immune adaptation.

## Data Availability

The mass spectrometry proteomics data have been deposited to the ProteomeXchange Consortium [35] via the PRIDE [36] partner repository with the dataset identifier PXD042097 (temporary reviewer account details: Username: reviewer_pxd075293@ebi.ac.uk Password: hsKSxG1W5D1s).

## Supplementary Materials

The following supporting information can be downloaded at: https://www.mdpi.com/article/10.3390/life16050753/s1.
