# Supplementary material for: Macrophage Proteomic Profiling Reveals Divergent TLR4-Dependent and -Independent Responses to Kdo2-Lipid A and Lipid IVa
Source: Life (Basel). 2026 May 1;16(5):753. doi: 10.3390/life16050753 (PMC13208710; doi:10.3390/life16050753)
Supplement: Supplementary file 1 [file life-16-00753-s001.zip › life-4280276-supplementary.pdf]

Supplementary Data

Macrophage Proteomic Profiling Reveals Divergent TLR4-Dependent and -Independent Responses to Kdo_2-Lipid A and Lipid IVa

Jiraphorn Issara-Amphorn[*1], Jenna Schoonmaker[*1], Clinton Bradfield[2], Sung Hwan Yoon[1], Iain D.C. Fraser[2], Aleksandra Nita-Lazar[1#]

[1] Functional Cellular Networks Section, Laboratory of Immune System Biology, National Institute of Allergy and Infectious Diseases, National Institutes of Health, Bethesda, MD 20892-1892, USA

[2]Signaling Systems Section, Laboratory of Immune System Biology, National Institute of Allergy and Infectious Diseases, National Institutes of Health, Bethesda, MD, USA;

*These authors contribute equally

[#] **Corresponding author:** nitalazarau@niaid.nih.gov

Supplementary Table S1 List of primers used in Real-Time PCR experiment

Name	Sequence (5′-3′)
M_TNF_F	CGTCAGCCGATTTGCTATCT
M_TNF_R	CGGACTCCGCAAAGTCTAAG
M_IL6_F	AGTTGCCTTCTTGGGACTGA
M_IL6_R	TCCACGATTTCCCAGAGAAC
M_IL10_F	CCAAGCCTTATCGGAAATGA
M_IL10_R	TTTTCACAGGGGAGAAATCG
M_Arg-1_F	GTGAAGAACCCACGGTCTGT
M_Arg-1_R	CTGGTTGTCAGGGGAGTGTT
M_iNOS_F	CACCTTGGAGTTCACCCAGT
M_iNOS_R	ACCACTCGTACTTGGGATGC

Supplementary Table S2 List of antibodies used in western blot experiment

Antigen (origin)	Dilutions	Company	Catalogue Number
Caspase 11 (Mouse)	1:1000	Cell Signaling Technology	14340
Beta-actin (Rabbit)	1:1000	Cell Signaling Technology	4970S
Secondary antibodies			
goat anti-rat IgG-HRP	1:50000	Santa Cruz	SC-2032
Mouse anti-rabbit IgG-HRP	1:25000	Santa Cruz	SC-2357

Supplementary Table S3 List of gene abbreviations and corresponding full names used in the main text.

Gene Name	Protein Name
ACOD1	Acyl-CoA desaturase 1
ARG1	Arginase-1
ATP5F1B	ATP synthase F(1) complex catalytic subunit beta, mitochondrial
CCL2	C-C motif chemokine 2
CEBPB	CCAAT/enhancer-binding protein beta
CMPK2	UMP-CMP kinase 2, mitochondrial
CS	Citrate synthase, mitochondrial
DLAT	Dihydrolipoyllysine-residue acetyltransferase component of pyruvate dehydrogenase complex, mitochondrial
ETFA	**Electron transfer flavoprotein subunit alpha, mitochondrial**
GBP2	Guanylate-binding protein 2
GBP3	Guanylate-binding protein 3
GBP7	Guanylate-binding protein 7
IDH2	Isocitrate dehydrogenase [NADP], mitochondrial
IDH3A	Isocitrate dehydrogenase [NAD] subunit alpha, mitochondrial
IDH3G	Isocitrate dehydrogenase [NAD] subunit gamma 1, mitochondrial
IFIT1	Antiviral innate immune response effector IFIT1
IFIT2	Interferon-induced protein with tetratricopeptide repeats 2
IFIT3	Interferon-induced protein with tetratricopeptide repeats
IL10	Interleukin-10
IL-1α	Interleukin-1 alpha
IL-1β	Interleukin-1 beta
IL6	Interleukin-6
IRGM2	Immunity-related GTPase family M protein 2
ISG15	Ubiquitin-like protein ISG15
ISG20	Interferon-stimulated gene 20 kDa protein
MALT1	Mucosa-associated lymphoid tissue lymphoma translocation protein 1
MDH2	Malate dehydrogenase, mitochondrial
NLRP3	NACHT, LRR and PYD domains-containing protein 3
NOS2 (iNOS)	Nitric oxide synthase, inducible
OASL1	2'-5'-oligoadenylate synthase-like protein 1
PTGH2	Prostaglandin G/H synthase 2
SOD2	Superoxide dismutase [Mn], mitochondrial
STAT1	Signal transducer and activator of transcription 1
STAT2	Signal transducer and activator of transcription 2
TNF	Tumor necrosis factor

Figure S1

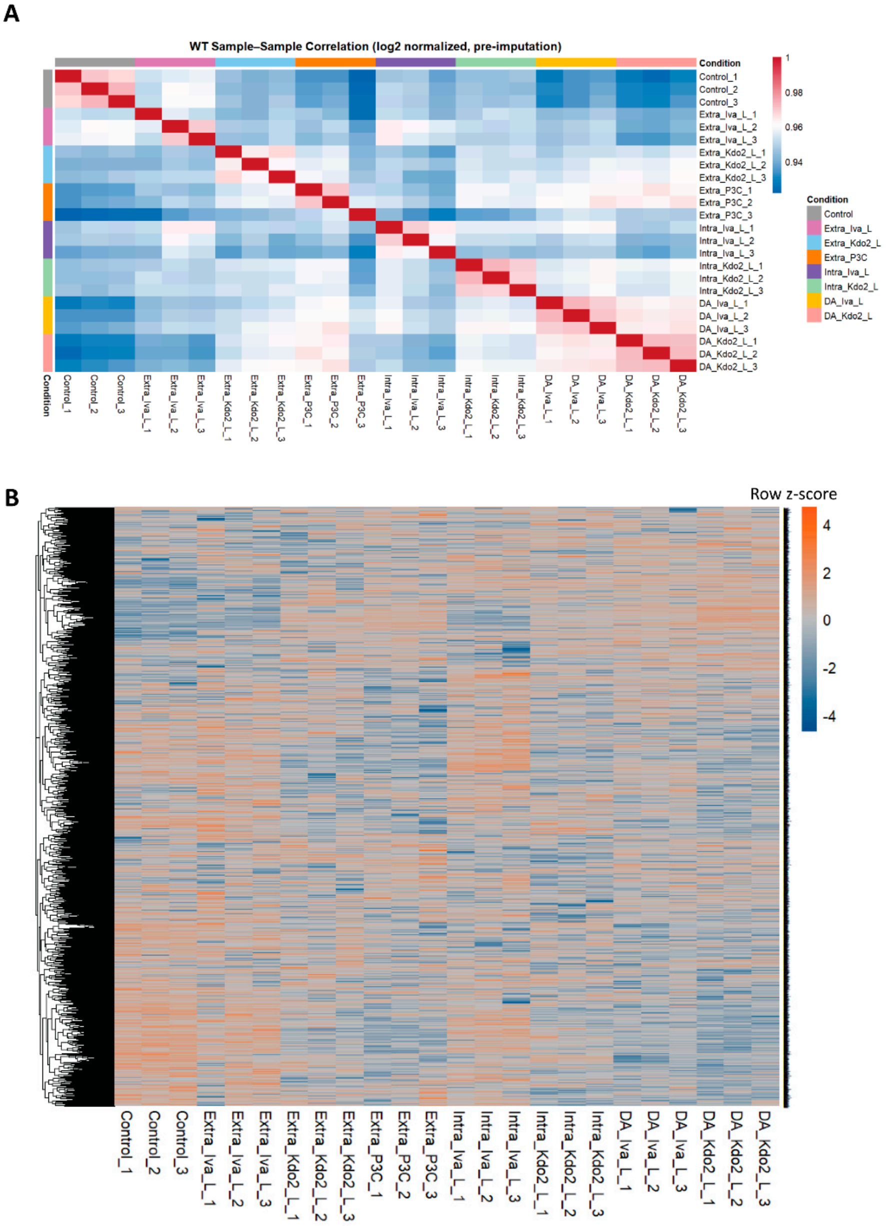

Figure S1. Quality control assessment across all conditions.
(A) Correlation heatmap based on Pearson's correlation coefficients across all samples and conditions. Colors represent the strength of correlation (0–1).
(B) Heatmap showing protein identification across all conditions.

Figure S2

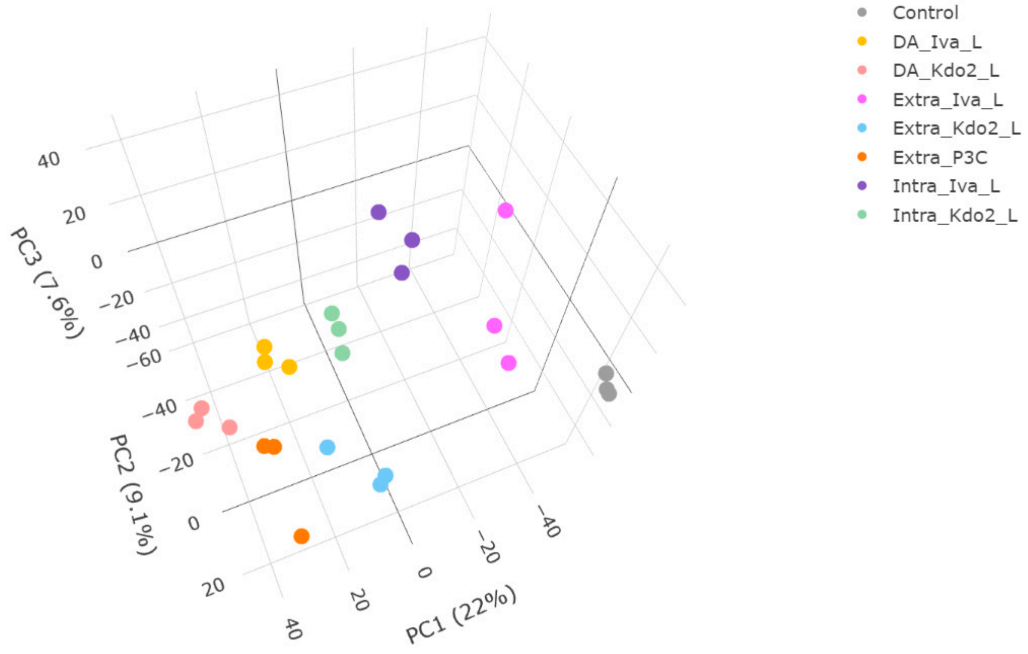

Figure S2. Three-dimensional principal component analysis reveals separation across conditions

Figure S3

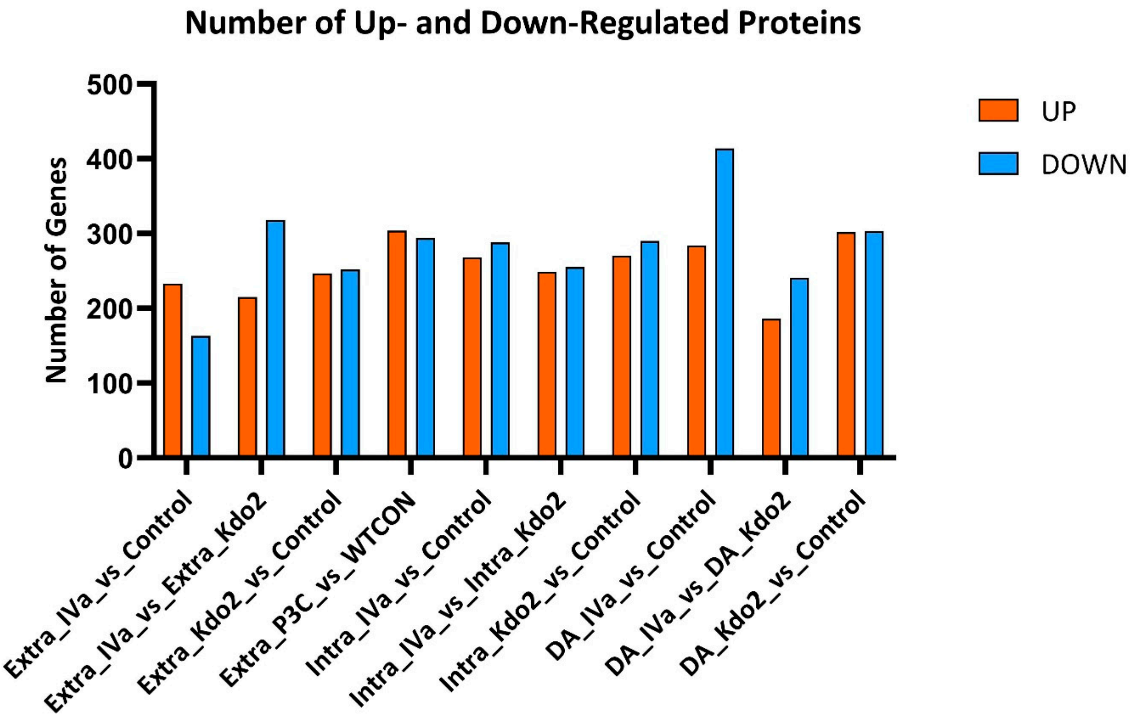

Figure S3. Numbers of up- and down-regulated proteins across conditions
